# Genetic modification to improve disease resistance in crops

**DOI:** 10.1111/nph.15967

**Published:** 2019-07-11

**Authors:** H. Peter van Esse, T. Lynne Reuber, Dieuwertje van der Does

**Affiliations:** ^1^ 2Blades Foundation 1630 Chicago Avenue Evanston IL 60201 USA; ^2^ The Sainsbury Laboratory University of East Anglia Norwich Research Park NR4 7UH UK

**Keywords:** biotechnology, food security, genetic modification, plant disease, plant pathogens, resistance

## Abstract

Plant pathogens are a significant challenge in agriculture despite our best efforts to combat them. One of the most effective and sustainable ways to manage plant pathogens is to use genetic modification (GM) and genome editing, expanding the breeder's toolkit. For use in the field, these solutions must be efficacious, with no negative effect on plant agronomy, and deployed thoughtfully. They must also not introduce a potential allergen or toxin. Expensive regulation of biotech crops is prohibitive for local solutions. With 11–30% average global yield losses and greater local impacts, tackling plant pathogens is an ethical imperative. We need to increase world food production by at least 60% using the same amount of land, by 2050. The time to act is now and we cannot afford to ignore the new solutions that GM provides to manage plant pathogens.

**Table:** 

Contents		
	Summary	70
I.	Introduction	70
II.	Intervention based on pathogen recognition and effectors	73
III.	Intervention by modification of defence signalling and regulation	74
IV.	Intervention by targeting recessive traits/susceptibility genes	75
V.	Intervention via other dominant plant resistance genes	75
VI.	Intervention with antimicrobial peptides	76
VII.	Intervention using RNA interference	77
VIII.	Practical path to deployment	77
IX.	Path to market in Africa	78
X.	How to deploy resistance durably	79
XI.	Trends that shape the future	80
XII.	The time is now	81
	Acknowledgements	81
	References	81

## I. Introduction

**Figure 1:**
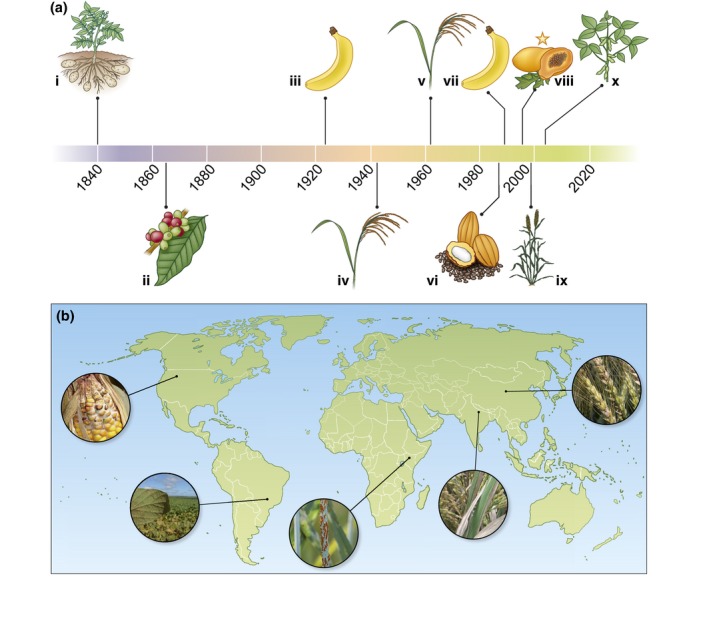
Major disease outbreaks in the last 150 yr and current critical disease challenges. (a) A timeline of major disease outbreaks: (i) Introduction of the oomycete *Phytophthora infestans* which causes potato late blight results in the Irish potato famine in which 1 million people die and 1.5 million people emigrate. (ii) The rust fungus *Hemileia vastatrix* wipes out the coffee crop in Sri Lanka; the British become tea drinkers. (iii) The vascular fungal pathogen causing *Fusarium* wilt of banana nearly wipes out the Gros Michel variety; the resistant Cavendish banana is adopted. (iv) The fungus *Cochliobolus miyabeanus*, which causes Brown spot disease of rice is a factor in the Great Bengal Famine in which 2 million people die of starvation. (v) Bacterial leaf blight of rice (*Xanthomonas oryzae* pv. *oryzae*) causes epidemics throughout Southeast Asia with yield losses up to 80%. (vi) Witches’ broom caused by the fungus *Moniliophthora perniciosa* is causing losses of up to 75% of annual cacao production in Brazil. (vii) The new *Fusarium* wilt isolate TR4 is identified and threatens Cavendish banana. (viii) Ringspot virus devastates the papaya industry in Hawaii; a GM variety is introduced that resists infection. (ix) A new race of the stem rust fungus *Puccinia graminis* (UG99) is spreading throughout Africa and the Middle East, threatening the world wheat supply. (x) Asian soybean rust caused by *Phakopsora pachyrhizi* reaches Brazil, costing growers US$2 billion annually in damages and control measures. (b) Examples of current disease challenges in major agricultural regions in the world that cause significant losses such as corn stalk and ear rots in the USA (4.15%), Soybean rust in Brazil (6.65%), Stem rust of wheat in sub‐Saharan Africa (8.89%), bacterial blight of rice in India (8.51%) and *Fusarium* head blight of wheat in China (8.75%). Source: Savary *et al*. (2019). Pictures: *Gibberella zeae* (corn ear rot) (photograph by Scot Adams, via Flickr, CC BY 2.0); *Phakopsora pachyrhizi* (Asian soybean rust) (photograph by Peter van Esse); *Puccinia graminis* f.* *sp.* tritici* (Wheat stem rust) (Photo by Yue Jin); *Xanthomonas oryzae* f. sp. *oryzae* (bacterial blight) (photograph provided by IRRI under creative commons licence); *Fusarium graminearum* (*Fusarium* head blight) (photograph by Gary C. Bergstrom, Cornell University, USA).

From the earliest days of farming, plant disease and pests have been a critical challenge for farmers. Although mankind has split the atom, travelled to the moon and connected the world, plant pathogens continue to be a significant challenge to food security despite our best efforts to thwart them (Fig. 1). Estimates of average global losses to diseases and pests range from 11–30% (Oerke & Dehne, 2004; Savary *et al*., 2019). Importantly, crop losses are highest in regions that already suffer from food insecurity (Savary *et al*., 2019). Losses from diseases would be far worse without past steady advances in agricultural practices, including cultural controls, agrochemical use and plant breeding. However, we have learned that there are no ‘silver bullets’. An integrated approach is needed to combat plant diseases, combining the best technologies and practices that are available.

The benefits of an integrated approach can be seen in the management of stem rust in wheat, a disease that caused periodic costly epidemics in the USA between 1918 and 1960 (Pardey *et al*., 2013). Only the combined effort of cultural practices (removal of barberry, the sexual host of this pathogen), improved chemical control (development of demethylation inhibitor and quinone outside inhibitor fungicides) and an extensive breeding program spearheaded by Norman Borlaug have enabled the containment of this particular disease of wheat.

However, there are limitations to such efforts. Some pesticides are rapidly losing efficacy due to pathogen evolution, and their use faces increasingly strict regulations to minimize unwanted side effects (Geiger *et al*., 2010; Bolton *et al*., 2012; Lamichhane *et al*., 2015; Wieczorek *et al*., 2015; Godoy *et al*., 2016; Berger *et al*., 2017). Crop breeding can produce resistance to individual diseases, but it is challenging to select for genetic resistance against multiple diseases simultaneously while maintaining the strong performance traits of elite varieties. For example, wheat blast is an emerging disease that will require wheat breeders to select for blast resistance while maintaining resistance against stem rust (Islam *et al*., 2016). To make matters more complicated, new races of stem rust have emerged and must also be tackled to ensure the stability of the world's wheat supply (Singh *et al*., 2015). Finally, the introgression of a single resistance via classical breeding facilitates pathogen adaption to that resistance.

The disease issues of wheat are not an isolated example, and challenges such as these are becoming more frequent as global warming and increased global trade facilitate the spread of known and emerging pathogens (Bebber *et al*., 2014). Top of these issues is the fundamental reality that 821 million people do not have enough to eat (FAO *et al*., 2018). The world population is projected to reach nearly 10 billion in 2050 (United Nations, 2017). This forecast brings with it the associated need to increase world food production by at least 60% (Alexandratos & Bruinsma, 2012; United Nations, Department of Economic and Social Affairs, Population Division, 2017) With this development in mind, tackling plant pathogens is not a mere academic exercise but an ethical imperative that requires action.

One of the most effective and sustainable ways to manage plant pathogens is to use GM and genome editing to expand the genetic tools available to breeders. In this review, we present an inventory of the genetic disease solutions currently available for bacterial, viral, fungal and oomycete pathogens. We will highlight the success stories of the potential of GM technologies and will outline what is needed for the effective deployment and realisation of the benefits they offer. Examples of genetic disease solutions are listed in Table 1. We evaluate these examples in light of population growth and other challenges and describe the trends that will shape the future.

**Table 1:** Examples of genetic disease solutions currently available for bacterial, viral, fungal and oomycete pathogens.

Point of intervention	GM technology	Example	References
Pathogen perception	Interspecies transfer of PRRs	EF‐Tu receptor (EFR)	Lacombe *et al*. (2010); Schoonbeek *et al*. (2015); Schwessinger *et al*. (2015); Boschi *et al*. (2017); Kunwar *et al*. (2018)
	Interspecies transfer of NLRs	**Rpi‐Vnt1**	Foster *et al*. (2009); http://www.isaaa.org/
		Bs2	Horvath *et al*. (2012)
	Modification of NLRs	Pikp‐1	Maqbool *et al*. (2015)
	NLR protease trap	PBS1 kinase	Kim *et al*. (2016)
	NLR resurrection	NRCs (NLR helpers)	Wu *et al*. (2017)
Pathogen effector binding	Deletion of effector targets	MAPK3K StVIK1	Murphy *et al*. (2018)
	Modification of effector binding sites	COI1	Zhang *et al*. (2015)
	Deletion of effector binding sites	*Os11N3/OsSWEET14*	Li *et al*. (2012)
	Addition of effector binding sites	*Xa27*	Hummel *et al*. (2012)
Defence signalling pathway	Altered expression of signalling components	NPR1	Xu *et al*. (2017)
	Altered expression of transcription factors	IPA1/OsSPL14	Wang *et al*. (2018b)
Recessive resistance alleles	Gene deletion	*mlo*	Kusch & Panstruga (2017)
	Gene modification	*bs5*	Iliescu *et al*. (2013)
Dominant plant resistance proteins	Interspecies transfer of signalling components	PFLP	Huang *et al*. (2007); Namukwaya *et al*. (2012); J. N. Tripathy *et al*. (2014); Tang *et al*. (2001); Huang *et al*. (2004); Ger *et al*. (2014); Yip *et al*. (2007); Liau *et al*. (2003)
	Transfer of detoxifying enzymes targeting pathogen toxins	Oxalate oxidase	Donaldson *et al*. (2001); Schneider *et al*. (2002); Hu *et al*. (2003); Dong *et al*. (2008); Walz *et al*. (2008); Partridge‐Telenko *et al*. (2011)
	Transfer of adult plant resistance (APR) alleles	Lr34	Krattinger *et al*. (2016); Risk *et al*. (2013); Schnippenkoetter *et al*. (2017); Sucher *et al*. (2017); Rinaldo *et al*. (2017)
Antimicrobial compound production	Transfer of antimicrobials from plants	Rs‐AFP defensin	Jha & Chattoo (2010); Li *et al*. (2011)
	Transfer of antimicrobials from microorganisms or animals	Virus KP4	Clausen *et al*. (2000); Schlaich *et al*. (2006); Quijano *et al*. (2016)
	Expression of synthetic antimicrobials	MsrA1	Osusky *et al*. (2000); Rustagi *et al*. (2014)
RNAi	Viral gene silencing through RNAi	***Coat protein*** **or ** ***replicase domain*** **gene from Papaya ringspot virus**	Fitch *et al*. (1992); Ferreira *et al*. (2002); Ye & Li (2010); http://www.isaa.org/
		*AC1* from bean golden mosaic virus	Bonfim *et al*. (2007); http://www.isaaa/org
		***Coat protein*** **gene from plum pox virus**	Scorza *et al*. (2013); http://www.isaaa.org/
		*Coat protein* gene from potato virus Y^a^	Lawson *et al*. (1990); http://www.isaaa.org/
		Putative *replicase domain* or *helicase domain* gene from potato leaf roll virus^b^	Lawson *et al*. (2001); http://www.isaaa.org/
		***Coat protein*** **gene from cucumber mosaic cucumovirus, zucchini yellow mosaic potyvirus and watermelon mosaic potyvirus 2**	Tricoli *et al*. (1995); http://www.isaaa.org/
	Fungal and oomycete gene silencing through RNAi	*HAM34* or *CES1* gene of *Bremia lactucae*	Govindarajulu *et al*. (2015)

Examples that are currently in the market are shown in bold.

a NewLeaf Y^®^ potato, no longer commercially available.

b NewLeaf Plus^®^ potato, no longer commercially available.

## II. Intervention based on pathogen recognition and effectors

Research over the past 20 yr has led to an increasingly refined knowledge of the plant immune system and its surveillance capacity. It is able to distinguish ‘self’ from ‘nonself’ as well as perturbations of ‘self’ by monitoring the extracellular and intracellular environment (Jones & Dangl, 2006; Cook *et al*., 2015). However, pathogens can overcome this system in an evolutionary arms race, producing proteins and molecules called effectors that are used to suppress host immunity and manipulate the plant cell to facilitate colonisation (Cook *et al*., 2015; Uhse & Djamei, 2018). Effectors are secreted into the extracellular environment or delivered in an orchestrated way into the host. This process is often done via specialised mechanisms such as the type III secretion systems of bacteria, haustoria of fungi and oomycetes and the stylet of nematodes (Panstruga & Dodds, 2009; Galán *et al*., 2014; Bird *et al*., 2015; Espada *et al*., 2016; Deng *et al*., 2017; Lo Presti & Kahmann, 2017).

Plants have two main surveillance systems to detect pathogen incursions. One class of receptors, known as pattern‐recognition receptors (PRRs), monitors the extracellular environment for conserved pathogen molecules such as flagellin, the bacterial elongation factor Tu, and chitin (Gómez‐Gómez & Boller, 2000; Zipfel *et al*., 2006; Miya *et al*., 2007; Faulkner *et al*., 2013; Cao *et al*., 2014; Hind *et al*., 2016). This class also recognises extracellular effectors that increase pathogen virulence (Wang *et al*., 1996; Thomas *et al*., 1997; Rep *et al*., 2005; van den Burg *et al*., 2006; van Esse *et al*., 2007; Catanzariti *et al*., 2015; Pruitt *et al*., 2015), and has been recently reviewed (Boutrot & Zipfel, 2017).

Intracellular pathogen effectors are recognized by another class of receptors that make up a large family of proteins characterised structurally by a nucleotide binding site (NBS) and leucine‐rich repeats (LRR) that are known as NOD‐like receptors (NLR) proteins (Dodds & Rathjen, 2010; Jones *et al*., 2016). This large family is well characterised and can be divided into two major groups in plants by features at their N terminus: one set has a Toll/interleukin‐1 receptor‐like (TIR) domain and the other a coiled coil (CC) domain (Jones *et al*., 2016), which confer discrete signalling capacity. Some NLRs have integrated domains that resemble/contain effector targets such as heavy metal‐associated binding domains, WRKY domains and RPM1‐interacting protein 4 (RIN4) (Le Roux *et al*., 2015; Maqbool *et al*., 2015; Sarris *et al*., 2016). Finally, an additional layer of the NLR resistance network is emerging in Solanaceous plants, a clade of helper NLRs has been identified and that connect to several NLRs that detect pathogens (Wu *et al*., 2017).

In the ongoing evolutionary arms race, some pathogens use the plant's defences against itself by misdirecting the host immune system to produce an immune response to the wrong pathogen to maintain host susceptibility. For example, some bacterial pathogens hijack the Coronatine‐insensitive protein 1 (COI1) jasmonate receptor, rewiring defence responses to activate jasmonate responses and concomitantly suppress the more effective salicylic acid defence pathway (He *et al*., 2004). Similarly, the necrotrophic fungal pathogens *Stagonospora nodorum* and *Pyrenophora tritici‐repentis* activate an inappropriate cell death response benefiting the pathogen by triggering the NLR receptor Tsn1 (Faris *et al*., 2010).

**Figure 2:**
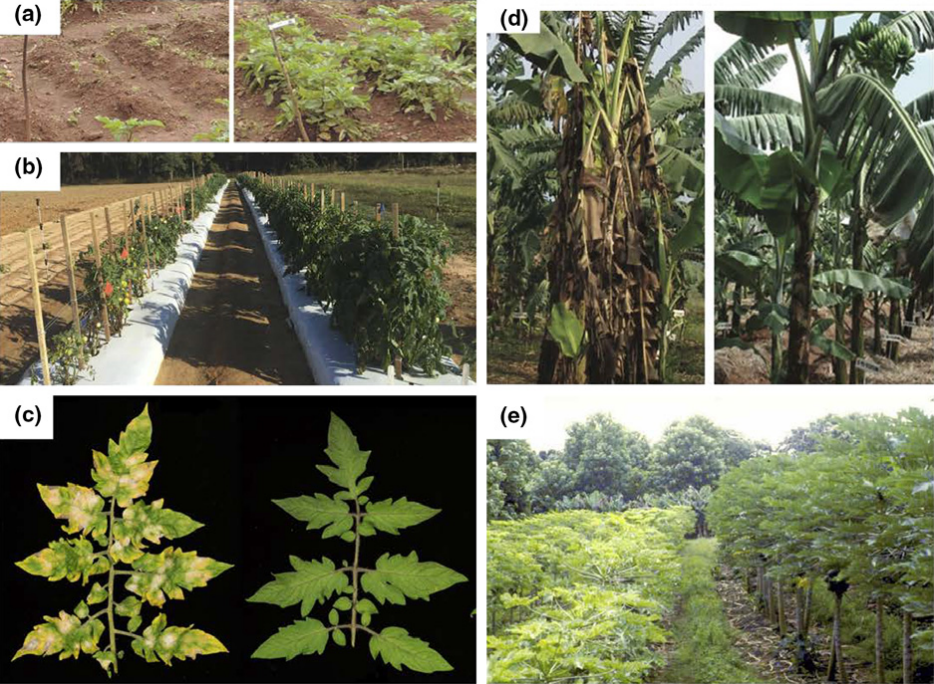
Success stories with different points of intervention: (a) The 3R potato contains three NLRs effective against *Phytophthora infestans*, which is present as a single mating type in Uganda and Kenya. (b) The cell‐surface EF‐Tu receptor (EFR) provides field level of resistance against the devastating tomato wilt pathogen *Ralstonia solanacearum*. (c) The Tomelo, genome‐edited tomato has resistance against powdery mildew due to modification of the *mlo* gene. (d) Heterologous expression of hypersensitive response‐assisting protein (Hrap) and plant ferredoxin‐like protein (Pflp) from sweet pepper provides field level resistance against *Xanthomonas* wilt disease in banana. (e) Overexpression of a virus coat protein in papaya provides commercial control against Papaya ringspot virus in Hawaii. In each case, the control plant(s) are on the left and the transgenic plants on the right. Pictures: photographs provided by (a) Marc Ghislain, © International Potato Center; (b) Dr Sanju Kunwar and Dr Mathews Paret, University of Florida; (c) Sophien Kamoun, The Sainsbury Laboratory. (d) Photograph reprinted by permission from Springer Nature Customer Service Centre GmbH: Springer Nature, *Nature Biotechnology*, field trial of *Xanthomonas* wilt disease‐resistant bananas in East Africa, (L. Tripathi *et al*., 2014). (e) Photograph provided by Dennis Gonsalves, republished with permission of the American Phytopathological Society, from Ferreira *et al*. (2002). Permission conveyed through Copyright Clearance Center, Inc.

Knowledge of the plant immune system has provided strategies to intervene at the point of pathogen perception. Extended or novel recognition capacity can be created in a number of ways, for example by introducing receptors from other plants with novel recognition specificity (Fig. 2a,b; Tai *et al*., 1999; Foster *et al*., 2009; Lacombe *et al*., 2010; J. N. Tripathy *et al*., 2014; Albert *et al*., 2015; Kawashima *et al*., 2016; Steuernagel *et al*., 2016; Witek *et al*., 2016; Ghislain *et al*., 2019); through modification of the integrated domains in NLRs that are targeted by the pathogen (Maqbool *et al*., 2015); or by reactivation of NLR genes disabled by effectors through the introduction of novel helper NLRs (Wu *et al*., 2017). Another original strategy is the design of the so‐called ‘NLR protease traps’. This strategy makes use of NLRs that can recognise the cleavage of plant proteins by specific pathogen proteases. This detection leads to a subsequent activation of immunity. Modification of the proteins monitored by such NLRs, such that the cleavage site will be targeted by a different pathogen protease, can broaden or alter the specificity of the plant's immune response (Kim *et al*., 2016).

Beyond strategies based on pathogen recognition, a growing understanding of effectors and their function has allowed interventions at the point of pathogen modulation of host responses. For example, knowledge of the plant targets of effector activity reveals which host components are manipulated to promote disease. This knowledge has been successfully applied to interfere with these points of vulnerability by removing them (Bozkurt *et al*., 2014; Boevink *et al*., 2016; Yang *et al*., 2016; Murphy *et al*., 2018) or replacing them with variants that are immune to effector action but retain the native function in the host (Zhang *et al*., 2015). For bacterial pathogens expressing transcription activator‐like (TAL) effectors that activate the expression of susceptibility genes in the host, resistance can be engineered by deletion of the TAL DNA binding sites in the promoter (Li *et al*., 2012; Jia *et al*., 2017). Another approach to engineer resistance to these bacterial pathogens is to add TAL effector binding sites to a cell‐death‐promoting (‘executor’) gene that is triggered by the TAL effectors present in common pathotypes (Hummel *et al*., 2012; Wang *et al*., 2018a).

Resistance of an entire plant species to all isolates of a microbial species is classically referred to as nonhost or species resistance. This nonhost resistance is brought about by physical factors, the plant immune system, and a general inability of the nonadapted pathogen to evade and/or disarm the plant's immune system (Nürnberger & Lipka, 2005). However, nonhost resistance does not represent a single phenomenon that can be used to engineer resistant crops. For example, most plant–pathogen systems cannot be neatly classified into the two extremes of host/nonhost systems (Bettgenhaeuser *et al*., 2014). In addition, there is no single mechanism behind nonhost resistance but various distinct and unique mechanisms (Cook *et al*., 2015). Therefore, the approaches that traditionally have been contained in the term nonhost resistance that are not perception‐related will be discussed in other sections of the review.

Resistance that is provided by NLRs and PRRs is robust, mechanistically well understood and, for NLRs, often results in strong immunity. There are clear advantages to working with the plant's innate immune system. Introduced receptors activate signalling pathways that are already in place in the plant. Importantly, activation of defences generally only occurs when a pathogen is perceived, minimising the cost to the plant overall. Furthermore, crop plants already contain hundreds of these receptors, therefore the likelihood that they are potential allergens or toxins is vanishingly small. Indeed, a late blight resistant potato containing an NLR receptor introduced from a wild relative is currently on the market in the USA (http://www.isaaa.org/). This is an important advance, but care must also be taken to deploy resistance genes durably; pathogens are extremely adaptive and single recognition specificities can be rapidly overcome by pathogen evolution.

## III. Intervention by modification of defence signalling and regulation

Perception of pathogens by the plant's immune system is translated into defence responses through hormones, signalling pathways and changes in defence genes. The major hormones involved in plant defences are salicylic acid (SA), jasmonate (JA), and ethylene (ET). In addition, there is extensive crosstalk with essentially all other hormonal signalling pathways, including gibberellins, auxin, brassinolide, cytokinins, and abscisic acid (reviewed by Robert‐Seilaniantz *et al*., 2011; De Vleesschauwer *et al*., 2014; Berens *et al*., 2017). Most major signalling components seem to be conserved throughout angiosperms (Berens *et al*., 2017), with some variations in the details of signalling, crosstalk, and mode of defence against different types of pathogens (De Vleesschauwer *et al*., 2014; Berens *et al*., 2017). In general, SA primarily mediates resistance to biotrophic pathogens, while JA in concert with ET mediates resistance to necrotrophic pathogens. There is cross‐inhibition between SA and JA resulting in tradeoffs between resistance to biotrophs and necrotrophs. Constitutive induction of SA or JA signalling produces resistance to pathogens ordinarily controlled by these responses but produces pleiotropic effects on growth and yield.

One way to engineer resistance without causing such pleiotropic side effects is to tightly control the timing and location of gene expression. An example of this strategy is the use of the TL1‐binding factor 1 (*TBF1*) promoter and leader sequences. *TBF1* contains two pathogen‐responsive upstream open reading frames to drive expression of either a constitutively active NLR protein or non‐expressor of pathogenesis‐related genes 1 (NPR1), a key regulator of SA response, in rice (Xu *et al*., 2017). The combined effects of transcriptional and translational control produced resistance to rice blast without a notable yield penalty.

A naturally occurring example of localised pathway overexpression is the quantitative resistance to biotrophic pathogens that is conferred by the loss of function of downy mildew resistance 6 (DMR6) in Arabidopsis (van Damme *et al*., 2008; Zeilmaker *et al*., 2015). *DMR6* is widely conserved and encodes a salicylate‐5‐hydroxylase that is induced around pathogen infection sites (Zhang *et al*., 2017). Loss of DMR6 function presumably increases the local SA concentration at the infection site (Zeilmaker *et al*., 2015). This knowledge was used to engineer a loss‐of‐function allele of a DMR6 homologue in tomato. This allele resulted in a quantitative resistance to biotrophic pathogens (de Toledo Thomazella *et al*., 2016).

Defence responses are controlled by networks of transcriptional regulators (Tsuda & Somssich, 2015). Therefore, the overexpression of specific transcription factors is a potential strategy to engineer resistance if pleiotropic effects on yield can be avoided. One interesting case is the rice gene *Ideal Plant Architecture 1 (IPA1)/OsSPL14* in which a natural allelic variant increased both yield and resistance to rice blast. Specific phosphorylation of the IPA1 protein in response to blast infection alters IPA1 binding specificity. This shift in specificity allows the protein to bind to and activate WRKY45, a defence regulatory transcription factor, providing quantitative resistance. By contrast, nonphosphorylated IPA1 promotes the expression of at least one yield‐related gene (Wang *et al*., 2018b). If this posttranslational regulation is conserved, *IPA1* expression may be useful to control disease in other crops.

## IV. Intervention by targeting recessive traits/susceptibility genes

Plant breeders have long been aware of recessive disease resistances, which have been identified in two ways, through mutagenesis and via breeding. With the onset of genome‐editing technologies, it is now possible to readily reconstitute recessive traits in other species. Many recessive traits can be generated by other methods in diploid crops, but genome editing opens up the possibility of reconstitution in polyploid crops such as wheat and potato. Most well understood recessive resistance traits remove or alter host factors needed for pathogen infection and hence are known as susceptibility genes. However, there are exceptions such as the *dmr6* mutation discussed above that alters signalling pathways. Recessive resistance can be very broad and durable, as exemplified by the powdery mildew resistance conferred by the mildew resistance locus O (*mlo*) allele, which is effective in crops as diverse as apple, tomato, barley and wheat (Kusch & Panstruga, 2017). For the complex wheat genome, all three homoeoalleles of *mlo* were targeted simultaneously using genome‐editing techniques (Wang *et al*., 2014). Alleles of *mlo* that give strong resistance unfortunately also give strong pleiotropic phenotypes (Kusch & Panstruga, 2017). However, the *mlo* allele can now be easily modified with gene‐editing tools. This process could allow a more precise calibration between achieving *mlo‐*mediated resistance and minimising *mlo‐*mediated pleiotropic effects (Fig. 2c; Nekrasov *et al*., 2017). Still, care should be taken with *mlo* modification because the allele may result in enhanced susceptibility to other pathogens. Known examples are the necrotrophic fungi *Magnaporthe oryzae*,* Fusarium graminearum and Ramularia collo‐cygni*, which all are more virulent in hosts with an *mlo* background (Jarosch *et al*., 1999; Jansen *et al*., 2005; McGrann *et al*., 2014). This increased susceptibility may be particularly relevant in wheat, in which blast disease caused by *Magnaporthe oryzae* pathotype *Triticum* is a critical emerging pathogen (Islam *et al*., 2016).

Another widely deployed recessive resistance that has potential value as a genome‐editing target is potyvirus resistance mediated by variants of eukaryotic translation initiation factor 4E (eIF4E). This type of resistance was first observed in mutants of *Arabidopsis thaliana* that exhibited loss of susceptibility to tobacco etch virus (TEV; *Potyvirus*) due to a deficiency in the *eIFiso4E* gene, an isoform of *eIF4E* (Lellis *et al*., 2002). Similar to *A. thaliana*, eIF4E‐mediated resistance against potyviruses is found in several resistant crop cultivars including pepper (*Capsicum annuum*), lettuce (*Lactuca sativa*), and wild tomato (*Solanum habrochaites*) (Ruffel *et al*., 2002, 2005; Nicaise *et al*., 2003). However, the plasticity in editing eIF4E appears to be restricted, because simple knockouts often result either in severe pleiotropic effects or a lack of effect due to redundancy (Bastet *et al*., 2017). Therefore, editing of eIF4E may be more successful when guided by naturally existing allelic variation (Bastet *et al*., 2017). Another example of a naturally occurring recessive resistance allele is bacterial spot 5 (*bs5*), which was identified in pepper breeding populations as a *Xanthomonas* resistance locus (Jones *et al*., 2002). The basis of resistance is a six base pair deletion in *Bs5*, a CYSTM protein, resulting in a protein product that lacks two amino acids in a highly conserved domain (Iliescu *et al*., 2013). Knockout mutations of CYSTM proteins give rise to severe growth and reproduction defects (Albert *et al*., 2015). This situation suggests that the specific change in *bs5* preserves other housekeeping functions and selectively interferes with pathogen action. *Bs5* is widely conserved, raising the possibility that the *bs5* phenotype may be recapitulated by creating the specific six base pair deletion in other plants susceptible to *Xanthomonas*, such as tomato.

Forward genetic approaches have yielded only a few targets for modification without incurring strong pleiotropic phenotypes in crops. Furthermore, recessive traits are typically not favoured by breeders, and therefore few have been molecularly characterised. The best and most widely deployed traits have been identified from nature. We therefore predict that the most effective recessive resistance traits will be those inspired by naturally occurring variants found in older breeding populations or wild relatives.

## V. Intervention via other dominant plant resistance genes

### V..1 Plant ferredoxin‐like protein and hypersensitive response‐assisting protein

Two interesting examples of plant proteins that confer disease resistance in various crops in a dominant fashion are plant ferredoxin‐like protein (PFLP) (Lin *et al*., 1997; Dayakar *et al*., 2003) and hypersensitive response‐assisting protein (HRAP) (Chen *et al*., 1998, 2000). Both proteins were isolated from sweet pepper (*Capsicum annuum*) and enhanced the production of reactive oxygen species and the hypersensitive response in reaction to harpins produced by Gram‐negative bacteria (Choi *et al*., 2013). HRAP may act in the extracellular space, where it could contribute to dissociation of harpins into active monomers or dimers, facilitating recognition by the plant (Chen *et al*., 1998, 2000). PFLP, formerly called amphipathic protein 1 (AP1), shows high similarity to ferredoxin proteins that function as electron carriers in photosynthetic tissues, where they are involved in many metabolic processes (Lin *et al*., 1997; Dayakar *et al*., 2003). Both PFLP and HRAP are effective against multiple bacterial pathogens when overexpressed in rice, banana, and other species (Tang *et al*., 2001; Ger *et al*., 2002, 2014; Liau *et al*., 2003; Huang *et al*., 2004, 2007; Pandey *et al*., 2005; Yip *et al*., 2007; Tripathi *et al*., 2010; Namukwaya *et al*., 2012; L. Tripathy *et al*., 2014). Field trials conducted in Uganda with *PFLP*‐ and *HRAP*‐expressing bananas indicated that both genes are highly effective against bacterial wilt caused by *Xanthomonas campestris* (Fig. 2d), while no negative effect on yield or plant morphology was observed (J. N. Tripathi *et al*., 2014, 2017). In addition, a bioinformatic approach did not reveal any potential allergenicity or toxicity associated with either of these proteins (Jin *et al*., 2017). A combination of *PFLP* or *HRAP* did not have a synergistic or additive effect, yet resistance in bananas that express both genes may be more durable (Muwonge *et al*., 2016).

PFLP and HRAP are valuable tools to engineer resistance to bacterial pathogens. The lack of mechanistic insights makes it difficult to predict what the full and long‐term effect of these proteins could be on plant health and agronomic performance. Additionally, the effect of overexpression of these genes on the performance of fungal, viral or oomycete pathogens has not been investigated. However, the urgent need to find a solution against bacterial wilt of banana, combined with successful field trials in which no negative effects were observed, argue for a staggered deployment combined with detailed monitoring of performance of HRAP and PFLP in the field.

### V..2 Detoxification enzymes

Plant enzymes that neutralise fungal toxins can play a role in plant defences, and transfer of their genes can improve resistance (Johal & Briggs, 1992). For example, *Fusarium* head blight is a significant fungal disease of wheat, as well as a source of mycotoxins in food that can poison humans and animals. Expression of a barley UDP‐glucosyltransferase in wheat metabolises the *Fusarium graminearum* toxin deoxynivalenol to a less toxic derivative, leading to reduced symptoms of *Fusarium* head blight in the field (Li *et al*., 2015). Similarly, oxalic acid is a virulence factor for *Sclerotinia sclerotiorum*, and transfer of oxalate oxidase from wheat produces significant resistance to *Sclerotinia* in many species, including peanut, tomato, potato, oilseed rape and soybean (Donaldson *et al*., 2001; Schneider *et al*., 2002; Hu *et al*., 2003; Dong *et al*., 2008; Walz *et al*., 2008; Partridge‐Telenko *et al*., 2011).

### V..3 Wheat adult plant resistance genes

The adult plant resistance (APR) or ‘slow rusting’ genes of wheat are another class of potentially transferable resistance genes. These genes produce dominant partial resistance to multiple biotrophic pathogens in mature plants but not in seedlings. Several APR genes are known, but only two, *Lr34* and *Lr67*, have been cloned. *Lr34* encodes an ATP‐binding cassette (ABC) transporter with an unknown substrate. The resistance allele in the D genome contains two specific mutations and is dominant over the other native Lr34 alleles in hexaploid wheat (Krattinger *et al*., 2009). Wheat lines carrying *Lr34* are partially resistant to multiple biotrophic pathogens including stem rust, stripe rust, leaf rust and powdery mildew. As a consequence, *Lr34* has been widely used in breeding. Similarly, the wheat *Lr67* resistance gene is a specific dominant allele of a hexose transporter that provides resistance to multiple rusts and powdery mildew. The protein encoded by the *Lr67* resistance allele is inactive in sugar transport, so it is likely to have a dominant negative effect (Moore *et al*., 2015). Introduction of the *Lr34* resistance allele by transformation into rice (Krattinger *et al*., 2016), barley (Risk *et al*., 2013), sorghum (Schnippenkoetter *et al*., 2017), maize (Sucher *et al*., 2017) and durum wheat (Rinaldo *et al*., 2017) and of *Lr67* to barley (Milne *et al*., 2018) also produced resistance to biotrophic pathogens. As for *mlo*, the mechanism by which resistance is triggered by *Lr34* and *Lr67* is poorly understood, although it is likely to involve the induction of biotic or abiotic stress responses that precondition the host to limit pathogen growth. Expression of these genes in some heterologous plants, for example *Lr34* in barley (Risk *et al*., 2013), has produced deleterious effects while, in other cases for example *Lr34* in durum wheat (Rinaldo *et al*., 2017), no obvious negative phenotypes were noted. Given the likely dominant negative mode of action of these proteins, relative quantities of wild‐type vs mutant proteins may need to be optimised in each system. This situation may also suggest that these types of resistances are more applicable to polyploid crops than diploid crops.

## VI. Intervention with antimicrobial peptides

Over the past decades, antimicrobial peptides and proteins have received a lot of attention as potential tools to create disease‐resistant crops. Antimicrobials are produced by organisms across all kingdoms and are a part of their innate immune systems (Brogden, 2005). Their activity is quite diverse and includes destruction of fungal cell walls, membrane permeabilisation, transcriptional inhibition and ribosome inactivation (Dempsey *et al*., 1998; van der Biezen, 2001; Brogden, 2005). Crops have been designed that express or over‐express (1) plant‐derived compounds such as pathogenesis‐related (PR) proteins and defensins that are normally produced during the plant's defence response, (2) antimicrobial proteins or peptides derived from microorganisms or animal cells, or (3) synthetic peptides designed based on sequences of existing antimicrobial compounds (Dempsey *et al*., 1998; van der Biezen, 2001; Castro & Fontes, 2005; Montesinos, 2007; Ali *et al*., 2018). Unlike the success of crops expressing anti‐insecticidal proteins from *Bacillus thuringensis* (Bt) that have been commercialised in different countries around the world, no crops expressing antimicrobial proteins have been commercialised to date (http://www.isaaa.org/). Development of crops engineered to express antimicrobials is challenging as antimicrobial proteins can often have phytotoxic effects, lead to over‐activation of stress responses, resulting in undesired phenotypes such as negative yield impacts, or have adverse effects on human or animal health (Montesinos, 2007). However, careful design or selection of suitable antimicrobials, followed by assessment of the agronomic performances of the engineered crops as well as of the potential impact on human or animal health may yet yield potential new solutions to crop diseases.

## VII. Intervention using RNA interference

RNA interference (RNAi) was first discovered in plants as a mechanism to recognise and defend against nonself nucleic acids. In addition to this defensive role, RNAi is a fundamental mechanism for the regulation of endogenous genes. Initiation of RNAi production occurs after double‐stranded RNA or endogenous microRNAs are processed by Dicer‐like proteins. The resulting small interfering (si)RNAs can be recruited by Argonaute (AGO) proteins that recognise and cleave complementary strands of RNA, resulting in gene silencing. RNAi‐based resistance can be engineered against many viruses by expressing ‘hairpin’ structures, double‐stranded RNA molecules that contain viral sequences, or simply by overexpressing dysfunctional viral genes (reviewed in Rosa *et al*., 2018). Moreover, a single double‐stranded RNA molecule can be processed into a variety of siRNAs and thereby effectively target several viruses using one hairpin construct. While viruses fight back with proteins that inhibit the silencing machinery of plants, the use of RNAi has nonetheless been validated as a powerful strategy to control many plant viruses (e.g. Lawson *et al*., 1990; Tricoli *et al*., 1995; Ferreira *et al*., 2002; Bonfim *et al*., 2007; Scorza *et al*., 2013), as well as nematodes (Huang *et al*., 2006) and insects (Baum *et al*., 2007; Bolognesi *et al*., 2012). The impact of RNAi technology deployed as a GM solution against viruses is powerfully demonstrated by the ‘Rainbow papaya’ (Fig. 2e). Introduction of the Rainbow papaya averted a collapse of the Hawaiian papaya industry from a severe outbreak of Papaya ringspot virus in the 1990s (Ferreira *et al*., 2002; Gonsalves *et al*., 2004). Since its introduction, 20 years ago, the GM trait introduced into Rainbow papaya has provided a sustainable and effective control of the virus. A similar GM trait has been used to engineer virus‐resistant squash, which has an even longer commercial history (Tricoli *et al*., 1995).

Following on these successes, RNAi has been explored as a strategy to control fungi and oomycetes as well, and initial patent applications for methods to control fungi using RNAi were made as early as 2006 (Roberts *et al*., 2007). Fungicide target genes in the pathogen are obvious candidates for this approach, as disruption is known to be lethal. Indeed, significant effects have been observed in *Fusarium* species by targeting the cytochrome P450, family 51 (*Cyp51*) genes that underlie the azole fungicide target sterol 14α‐demethylase with host‐induced gene silencing (HIGS) (Koch *et al*., 2013). Additional pathogen genes that have been targeted include pathogenicity factors, developmental genes and genes involved in metabolism. HIGS of a *Verticillium* hydrophobin gene resulted in strong resistance to *V. dahliae* in cotton (Zhang *et al*., 2016). Similarly, HIGS targeted to a cellulose gene and a highly expressed conserved gene of *Bremia lactucae* resulted in high levels of resistance to this pathogen in lettuce (Govindarajulu *et al*., 2015). More often, however, HIGS experiments produce quantitative effects, for example when targeting rust fungi (Panwar *et al*., 2013, 2018; Yin & Hulbert, 2018) and virulence factors of *V. dahliae* in tomato (Song & Thomma, 2018). Overall, HIGS seems to be quite effective against some pathogens (Govindarajulu *et al*., 2015; Wang *et al*., 2016) but ineffective against others (Kettles *et al*., 2018). However, there appears to be an apparent disconnect between the earliest publications and patent filings on HIGS a decade ago and practical examples of HIGS deployed in the field. This may suggest that, although effects are observed, they are not strong enough to provide field level solutions to many pathogens.

Until recently, it was unclear how small RNA molecules would be exchanged between host and pathogens. However, compelling evidence has shown that small RNAs are delivered to fungal pathogens via extracellular vesicles (Cai *et al*., 2018). A better understanding of this process in diverse plant−pathogen interactions may allow us to better optimise HIGS strategies to provide field‐relevant levels of resistance. In short, RNAi appears to be a promising additional control strategy in the arsenal of plant breeders against at least some pathogens. The modular nature of RNAi is especially suitable to multiplexing via synthetic biology approaches. In addition, RNAi strategies may be particularly relevant when no pathogen resistance can be identified in natural populations.

## VIII. Practical path to deployment

After a solution against a crop disease is discovered in the laboratory, it must pass several further hurdles. The first of these hurdles is that it also must be effective in the field without reducing agronomic performance. Subsequently, a commercial development process requires the generation and evaluation of a large number of transgenic lines to choose a transgenic event that only has the specific and intended modifications. Once this rigorous vetting procedure has been completed, introgression of this event into commercial cultivars and development of a regulatory dossier is initiated (reviewed by Prado *et al*., 2014).

A genetically modified crop must meet regulatory approval in each country where it will be grown or imported. Regulatory requirements in different countries are not standardised, and this situation increases the complexity of the task (Prado *et al*., 2014). Costs are often prohibitive, with estimates for international product deregulation between US$7M and US$35M (Kalaitzandonakes *et al*., 2007; Phillips McDougall, 2011) out of a total estimated product development cost of US$136M (Phillips McDougall, 2011). A cost−benefit calculation is fundamental to determining the commercial practicality of different disease‐resistance solutions. As an example, Box 1 summarises the data needed to deregulate a transgenic disease‐resistant crop in the USA. In the USA, the Food and Drug Administration (US FDA) assesses evidence for the safety of any added protein and the substantial equivalence of the crop to its nontransgenic equivalent. The Environmental Protection Agency (US EPA) assesses the consumer safety and lack of environmental impact of any ‘plant incorporated protectant’. The United States Department of Agriculture (USDA) assesses the potential of the new plant to be a weed or plant pest. The level of evidence required for any of these points is determined by the relative risk of the introduced trait. As mentioned above, the first immune receptor has been deregulated in the US: the Rpi‐vnt1 receptor with effectivity against late blight of potato. In this case, the US EPA and FDA accepted arguments that the protein is present at vanishingly small amounts, is not a potential allergen, and is similar to proteins already consumed (Clark *et al*., 2014; FDA, 2015; EPA, 2017). Therefore, animal feeding studies and extensive biochemical analyses on purified protein, which would have been extremely difficult in the case of an NLR (Bushey *et al*., 2014), were not required. However, a hypothetical product that expressed high levels of an artificial antimicrobial protein without a history of safe consumption would require more extensive evidence for safety and have concomitantly higher regulatory costs. Given the costs, time, and risk involved in developing and deregulating GM crops, only very high‐value traits on broad acreage crops are currently commercially viable targets. Only a handful of crop diseases, for example soybean rust and potato late blight, meet this economic threshold.

Box 1 **Regulatory authorities and scope of regulation of bioengineered crops in the United States (EPA, 2019; FDA, 2019; USDA, 2019)**

1

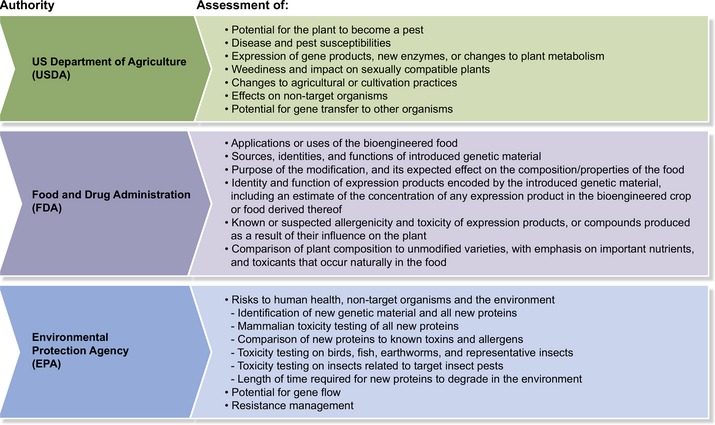

The USDA has recently released guidelines for the regulatory status of plants produced by gene editing, stating that certain classes of changes that could have been accomplished by traditional breeding are not subject to regulation if they were produced without plant pest sequences (that is not by *Agrobacterium* transformation). These changes include deletions, single nucleotide changes, and insertions of DNA from a sexually compatible relative (USDA, 2018). Although disease‐resistant food crops may still be subject to regulation by the FDA and EPA, this ruling drastically decreases the cost of bringing many types of disease‐resistant crops to market in the USA. By contrast with the scientifically based and pragmatic US guidelines, a recent ruling in the European Union states that all plants produced by gene editing are still subject to the same regulation as transgenic plants (Callaway, 2018). This effectively rules out the use of gene editing for any crop grown in or exported to Europe, robbing European growers of powerful solutions that could lead to more sustainable agriculture.

## IX. Path to market in Africa

Africa is the continent where there is the greatest need and opportunity for agricultural growth, given the expected population growth and amount of unused arable land. Genetically modified or edited crops could play a significant role in helping Africa's agriculture to meet the needs of its growing population. Currently, adoption of GM crops in Africa is limited as they are commercially available only in Sudan (cotton) and South Africa (maize, cotton and soybean) (ISAAA, 2017). The adoption of GM crops in those countries has been successful, and acreage has increased steeply since they were first introduced (ISAAA, 2017). At present, several other countries in Africa have regulatory frameworks in place and are conducting field trials to prepare for general release when government policies allow. These countries are Ethiopia, Kenya, Uganda, Tanzania, Mozambique, Malawi, Swaziland, Cameroon, Nigeria, Ghana, and Burkina Faso (ISAAA, 2017). In Uganda, field trials are being conducted with potato expressing a stack of immune receptors providing protection against potato late blight disease, as well as bananas that are resistant to bacterial wilt (Fig. 2a; L. Tripathy *et al*., 2014; Ghislain *et al*., 2019).

Regulatory costs and time associated with the process can vary greatly and depend on the crop, the country, the developer and the inserted genes. Cost estimates for the development of a single GM variety (late blight resistant potato) in a developing country by a not‐for‐profit institution vary from US$1.4 million to US$1.6 million over 8–9 yr of review (Schiek *et al*., 2016). In many African countries, genome‐edited crops are expected to be regulated through the GM regulatory framework, similarly to the laws in Europe (ISAAA, 2017).

In Africa, as elsewhere, a second major barrier to advancing genetically engineered disease resistance is public concern about the safety of GM crops, despite an overwhelming body of evidence for the safety of these crops (National Academies of Science, Engineering and Medicine, 2016). This situation is unfortunate, given the potential for GM to address food losses caused by plant disease. This, in turn, can help to increase food production locally to accommodate a rapidly growing population. Africa's close ties to Europe influence its attitudes about GM crops, which are generally conservative and not based on scientific facts. Given the challenges that lie ahead, a shift to a scientific and pragmatic stance on the use of GM technology would be timely. The success of adoption of GM crops in Sudan and South Africa and the ongoing trials and safety assessments in other African countries might convince the public and politicians to open the doors to these molecular breeding approaches.

## X. How to deploy resistance durably

It is clear that plant pathologists and breeders have uncovered a versatile arsenal of solutions to bring to bear against plant pathogens that offers great potential for global food security and sustainability. However, plant pathogens are highly adaptable and have much faster life cycles than their plant hosts, and therefore resistance conferred by most single genes or modes of action will be easily defeated. This reality is a key challenge for classical breeding, because durable resistance generally requires combinations of multiple resistance genes and quantitative trait loci (QTLs) at different locations in a genome. The problem is compounded by introgression of new resistances from non‐elite cultivars and wild relatives, which are often subject to yield loss due to linkage drag. Moreover, when a new disease or breeding goal appears, breeding for the new and existing traits becomes even more complex. Last, some important crop plants are notoriously difficult to breed, such as the tetraploid potato, sugarcane, and the (almost) sterile banana.

Genetic modification allows several dominant disease‐resistance genes to be introduced together in a single well‐characterised region of the genome overcoming many of these challenges. Critically, it is possible to introduce several dominant resistance traits into elite cultivars, polyploid crops, sterile plants and parental lines to be used in subsequent breeding efforts. Even if additional breeding is required, the key combined resistances will remain intact as a single locus. Unlike dominant resistance traits, recessive resistances present more of a challenge as they cannot be combined at a single locus, but genome editing in base breeding lines can accelerate the process of introducing these resistances.

Each resistance approach reviewed here took years of collaborative research effort. Many of the solutions were found by tapping into the large, but not unlimited, genetic diversity found in nature. It is therefore critical that thoughtful, durable deployment and stewardship of these hard‐won resources is achieved. The definition of durable resistance is fluid, and in each case is dependent on the strength of resistance required and the time that is needed for the resistance to hold (Brown, 2015). The question must be – ‘Does the combined solution work well enough and long enough?’

Given the requirement for clear resistance phenotypes in the field, many combined solutions will include the strong resistance conferred by NLR genes. Several factors influence the durability of combined NLR genes; major factors being the impact on virulence of the pathogen, the strength of the resistance, the exposure of a pathogen to an NLR, the total inoculum in the environment, and the capacity of the pathogen for sexual recombination (or lack thereof) (McDonald & Linde, 2002; Brown, 2015; Stam & McDonald, 2018). Although these factors are likely to play a role in the durability of each of the other resistance mechanisms reviewed here, the points of impact are likely to be different. Therefore, combining several modes of action will potentially result in resistance that is both effective and long lasting. For example, an NLR stack of Tm‐2^2^ and Tm‐2 is predicted to be durable, as the two mutations in the movement protein of tomato mosaic virus that are required to overcome this resistance are predicted to disrupt function of the viral movement protein (Lanfermeijer *et al*., 2005). However, even greater durability may be achieved by combining these two genes with a different mode of action such as a hairpin RNAi construct.

Both the private and public sectors should pursue ever more durable ways of using the agricultural resources at hand. In the long run, a shift away from environmental and genetic uniformity in agricultural systems will result in a more durable status quo between crop and pathogens (McDonald & Stukenbrock, 2016). However, a critical assessment needs to be made on the timelines that this would take and at what cost to the efficiency and productivity of monoculture‐based agriculture this change would come. Compared with an average of 13 yr to deploy new transgenic lines, it can be debated whether an overhaul of the agricultural system before the population peak of 2050 is desirable or even possible. The pragmatic approach is to work with the best possible solutions that we have available today to ensure we will be in a position to deploy even better solutions later this century.

## XI. Trends that shape the future

There are several trends that will affect the way in which we will design solutions and deploy traits. As exemplified in the paragraph above, it is important that several traits can be combined into one locus, preferably at a known location in the genome. This approach presents a unique technical challenge as cassettes need to be designed that contain multiple traits against one disease. An important trend therefore is the technical advance that is made to construct cassettes that contain multiple traits. Already, this is feasible to a certain extent, as has been demonstrated with gene stacks that contain three NLRs that recognise *P. infestans* (Ghislain *et al*., 2019, Fig. 2a) and a five *R* gene stack in wheat against wheat stem rust (Michael Ayliffe, personal communication). Although generating cassettes with multiple large inserts has traditionally been challenging, recent technical advances such as Gene Assembly in *A* *grobacterium* by Nucleic acid Transfer using Recombinase technologY (GAANTRY) has enabled the generation of stable cassettes with up to 10 genes with at total size of 28.5 kbp (Collier *et al*., 2018). Therefore, the generation of a cassette that can effectively target one or two key diseases is now technically feasible. As traits are dominant, combinations can subsequently be made via breeding. An example of what such a strategy may look like is the commercial maize line known by its trade name SmartStax™. To generate this line, four different biotech maize lines were crossed and resulted in the combination of six *Bt* genes and two herbicide tolerance genes, providing control for weeds and lepidopteran insects. Nonetheless, the ability to generate large stacks of combined traits will be a critical development over the coming years.

For gene stacks to be functional, the causal genes that underlie resistance must be identified. For many crops the reservoir of cloned resistance genes is still limited. However, the second trend is that new affordable sequencing technologies combined with bioinformatic approaches allow ever faster identification of causal resistance genes. This identification can now already be done, even in complex genomes such as wheat and potato and wild relatives of crops such as pigeonpea (Kawashima *et al*., 2016; Steuernagel *et al*., 2016; Witek *et al*., 2016). In addition, the ability to obtain a good quality reference genome assembly is now reduced to standard practice. With the ever‐dropping cost of sequencing and increase in processing power these approaches will soon become commonplace. This capability is important because it allows scientists to explore the rich genetic diversity of crop relatives. Nature has had millions of years to test and select resistance mechanisms, providing a wealth of potentially validated strategies. By making use of affordable, powerful sequencing capacity, wild germplasm can be mined for a distribution of resistance traits at the centre of origin. As many pathogens have co‐evolved with a wild progenitor species, a resistance trait against a specific disease that is overrepresented in the centre of origin of a wild progenitor may reflect that this trait is particularly effective with little cost to the host (Stam *et al*., 2017).

A third trend is the miniaturisation of sequencing technologies. Pathogen detection and analyses of the microbiome with a portable DNA sequencer has already successfully been executed (Hu *et al*., 2019). By the time that most solutions developed today will reach the field, such real‐time monitoring of pathogen populations in the field will be possible and likely standardised enough to be performed by growers or agronomists. A better understanding of pathogen population structure and dynamics may inform the best intervention strategy (genetic or other) against a given disease, for example via identification of key effectors.

No review would be complete without mentioning the fourth trend, which is the expanding use of genome‐editing tools. Genome editing can already be used to produce recessive traits, however as we set out in this review relatively few effective recessive targets have been identified. In addition, most targets that are simple knockouts have already been introduced via tilling, except in polyploid crops. Editing also provides the ability to precisely modify existing resistance genes or their expression, allowing the *in situ* conversion of a susceptible allele to a resistant one. Use of genome editing to integrate dominant resistance traits at a single locus will have even broader benefits, although it is important to note that this approach is already feasible using site‐specific recombination (SSR) systems (Srivastava & Thomson, 2016). However, the more efficient introduction of traits, or replacement of single traits in a stack may be accomplished via genome‐editing technologies (Rinaldo & Ayliffe, 2015). In addition, genes can be introduced anywhere in the genome. For instance, introducing new resistance genes next to already existing resistance loci could generate greater flexibility for the breeder. Gene stacks could be created and updated by precise addition and removal of genes. Finally, precise gene editing would introduce less ‘foreign’ DNA than *Agrobacterium* transformation, which may help deregulation in some countries. However, this is a legislative and not a scientific advantage.

A final trend that is developing in parallel is the rapid progress in protein structural biology techniques such as cryo‐EM. This will allow a better understanding of NLR and PRR function. Unlike the other trends described here, this trend has the capacity to be truly transformative in the way plant disease is tackled. The first step towards designing recognition specificity has already been made via the modification of HMA domains in NLRs with integrated domains (Maqbool *et al*., 2015). In addition, some NLR families can recognise multiple effectors from different pathogens via direct interaction (Saur *et al*., 2019). Unlike PRR proteins, how NLRs signal has been one of the long‐standing questions in plant pathology. However, two recent landmark publications have described the mechanism of activation for the *A. thaliana* HOP‐ACTIVATED RESISTANCE 1 (ZAR1) protein using cryo‐EM techniques (Wang *et al*., 2019a,2019b). All this information can be coupled to advances that are made in deep learning and synthetic biology, such those already used in drug discovery (Chen *et al*., 2018). This situation may enable scientists to develop recognition specificities for key pathogen effectors in the form of designer NLRs and PRRs.

## XII. The time is now

We have in hand the means to thwart plant diseases that have plagued mankind since the dawn of agriculture. The genetic methods to combat disease reviewed here are more effective, environmentally friendly and safer than many current, common methods of control. We need to double our food production in 50 yr, and 70% of this increase needs to be achieved by adopting new technology. Therefore, we cannot ignore these approaches. However, almost none of the currently available GM solutions have reached growers, in large part due to consumer anxieties, even though the most ardent opponents of the technology ironically are the least informed about science and genetics (Fernbach *et al*., 2019), and the scientific consensus is that GM crops are as safe as those developed by classical breeding (National Academies of Sciences & Engineering and Medicine, 2016). Unfortunately, some legislators ignore the facts about GM safety and benefits, therefore blocking solutions that would benefit society broadly (Court of Justice of the European Union, 2018). Due to global trade, Europe's conservative attitude towards GM crops has affected agriculture worldwide, including those regions where GM crops could have great local benefits. To break this deadlock, interdisciplinary approaches that include social scientists need to be taken, and scientists should stay in dialogue with consumers and policy makers. It is up to this generation of scientists, seed companies, international agricultural organisations, and legislators to responsibly deploy the valuable and available genetic disease solutions to help reduce the footprint of agriculture on the planet while increasing its output.
